# Microservice-Oriented Platform for Internet of Big Data Analytics: A Proof of Concept

**DOI:** 10.3390/s19051134

**Published:** 2019-03-06

**Authors:** Zheng Li, Diego Seco, Alexis Eloy Sánchez Rodríguez

**Affiliations:** Department of Computer Science, University of Concepción, Concepción 4070409, Chile; dseco@udec.cl (D.S.); alexisanchez@udec.cl (A.E.S.R.)

**Keywords:** big data analytics, Internet of Things, microservices architecture, microservice-oriented platform, software defined infrastructure

## Abstract

The ubiquitous Internet of Things (IoT) devices nowadays are generating various and numerous data from everywhere at any time. Since it is not always necessary to centralize and analyze IoT data cumulatively (e.g., the Monte Carlo analytics and Convergence analytics demonstrated in this article), the traditional implementations of big data analytics (BDA) will suffer from unnecessary and expensive data transmissions as a result of the tight coupling between computing resource management and data processing logics. Inspired by software-defined infrastructure (SDI), we propose the “microservice-oriented platform” to break the environmental monolith and further decouple data processing logics from their underlying resource management in order to facilitate BDA implementations in the IoT environment (which we name “IoBDA”). Given predesigned standard microservices with respect to specific data processing logics, the proposed platform is expected to largely reduce the complexity in and relieve inexperienced practices of IoBDA implementations. The potential contributions to the relevant communities include (1) new theories of a microservice-oriented platform on top of SDI and (2) a functional microservice-oriented platform for IoBDA with a group of predesigned microservices.

## 1. Introduction

The emerging age of big data is leading us to an innovative way of understanding our world and making decisions. In particular, it is the data analytics that eventually reveals the potential values of datasets and completes the value chain of big data. When it comes to big data analytics (BDA), in addition to theories, mathematics, and algorithms, suitable infrastructures and platforms are also prerequisites to efficient BDA implementations. In practice, it is the managed and scheduled resources that deliver computing power to data processing logics to fulfill BDA jobs at runtime. In this article, we clarify the physical resources to be infrastructure, while treating the intermediate supporting mechanisms as layered platforms between the runtime jobs and their infrastructure.

Driven by increasing BDA demands, various and numerous frameworks and tools have emerged as BDA platforms [1]. According to their functional specifications, these BDA platforms generally combine data processing logics together with computing resource management. For instance, to perform Hadoop MapReduce jobs, a Hadoop system including its distributed filesystem needs to be installed and configured on a dedicated cluster managed by YARN [2]. Consequently, the current BDA implementations still require significant effort on environmental configurations and platform manipulations.

Furthermore, based on the de facto platforms, BDA applications tend to be environmentally monolithic because they are essentially stuck to predefined computing resources no matter how redundant the resources are. This environmental monolith is generally acceptable for traditional BDA problems with centralized workloads. When it comes to the booming ecosystem of Internet of Things (IoT), there is no doubt that such a mechanism will require tremendously extra overhead for big data collection from largely distributed sensors/devices for later analytics [3]. Nevertheless, IoT-oriented data analytics could eventually become inefficient and expensive if we always transfer and process data cumulatively in a central repository, because many IoT-oriented BDA problems (which we name “IoBDA”) can be addressed without combining the originally distributed data [4,5].

Inspired by software-defined infrastructure (SDI) [6,7], we propose the use of a standard microservice-oriented platform to relieve the complexity in IoBDA implementations and to break the environmental monolith of IoBDA applications. In fact, it has been identified that deploying physical infrastructures for BDA applications can be costly and challenging [8], not to mention addressing the possibly tremendous heterogeneity and diversity in hardware. To reduce the infrastructural cost, as well as obtain deployment agility and automation, there has arisen a software-driven trend in making the computing environment programmable and software-defined [9]. Typical examples of SDI include software-defined network (SDN) and software-defined storage (SDS). SDN decouples the data transmission control from networking devices, such as switches and routers [10], SDS separates the data store management from storage systems [2], and both of them leverage heterogeneous hardware to facilitate support of workload demands via open-interface programming. In other words, SDI is mainly focused on the programmable management and control of computing resources. In contrast, our proposed microservice-oriented platform is expected to further isolate domain-specific business logics from the resource control and management.

This article mainly portrays the proof of concept of the microservice-oriented platform for IoBDA, and it demonstrates this idea by using Monte Carlo analytics and Convergence analytics that both fit in the characteristics of the IoT environment. We believe such an idea can theoretically be justified by the philosophy of osmotic computing [11] and technically be supported by the existing underlying frameworks [12]. We are still investigating IoT-friendly data processing logics and gradually migrating them to the microservices architecture (MSA) in order to further solidify our proposed platform.

On the basis of our ongoing efforts and current outcomes, we expect this article to make a twofold contribution to both the IoT and BDA communities:

- A new theory of a microservice-oriented platform on SDI. The theory essentially evolves the architecture of BDA implementations in general, i.e., further decoupling data processing logic from computing resource management. Such an architecturally loose coupling will generate more research opportunities in academia and will better guide BDA practices, particularly in the dynamic IoT environment.
- A functional microservice-oriented platform with predesigned microservices. In addition to facilitating IoBDA implementations for practitioners, a functional platform will, in turn, drive its own evolution along two directions. Vertically, our use cases can help validate and improve the underlying technologies (deeper-level platforms or frameworks) [12] while strengthening their compatibility with our platform. Horizontally, the initial functionalities can inspire more research efforts that aim to enrich microservice-oriented data processing logics and expand the applicability of this platform.

The remainder of this article is organized as follows. Several studies related to our work are summarized in Section 2. Section 3 elaborates on the definition of the microservice-oriented platform for IoBDA and briefly justifies its feasibility. Section 4 and Section 5 demonstrate two IoT-friendly data processing logics to facilitate implementing Monte Carlo analytics and Convergence analytics, respectively, which will, in turn, be used to prototype our microservice-oriented platform. Section 6 draws conclusions and outlines two directions for our future work.

## 2. Related Work

By offering many benefits, including better scalability, reliability, and maintainability, microservice technologies have been employed successfully in practical projects (e.g., data organization and analysis in a Solar Nexus project [13]). However, these projects are still based on a dedicated computing environment, and their application scenarios have nothing to do with distributed data sources in the dynamic IoT environment.

On the basis of observations that recent technological advances are moving Cloud’s centralized computing capacity close to users, osmotic computing has been proposed to represent the new paradigm of dynamic microservice-oriented computing that exploits both Cloud and Edge resources [11]. Unlike our proposed platform in this article that aims at technical solutions to IoBDA, the authors of [11] essentially supply a philosophy of MSA in smart environments. On the other hand, this philosophy can be used to justify the novelty and feasibility of our proposed microservice-oriented platform.

As a matter of fact, given its advantages of agility and scalability, MSA has been considered promising middleware architecture for IoT in general [14]. When it comes to the BDA domain, the closest work to ours is the distributed BDA framework for IoT [12]. It also emphasizes handling different data streams from various sources. Nevertheless, the layered framework in this study is mainly a microservice management mechanism including management policies, a messaging infrastructure, etc. Although this mechanism is supposed to facilitate the development of MSA-based applications, it does not care about the design and implementation of individual microservices, not to mention their boundary identification. On the contrary, our work is intended to support specific data processing logics with predesigned standard microservices (and/or templates), which will largely help inexperienced developers facing immature IoBDA implementations. Considering the microservice management at runtime, in particular, this framework [12] can act as a supporting layer beneath our proposed platform.

## 3. Conceptual Explanation and Justification

Emerging from the agile practitioner communities, MSA emphasizes addressing the shortcomings of monolithic architecture by decomposing the monolith into multiple small-scale, loosely coupled, and independently deployable microservices and making them communicable with each other through lightweight mechanisms (e.g., REST APIs) [15]. In fact, the booming container technology has enabled virtualization for many types of edge devices [16], and this eventually supports building container-based microservices within those devices. Note that it is possible to introduce agent microservices (similar to the concept of “infrastructure mediate systems” [17]) to represent or coordinate non-virtualizable binary sensors.

Unfortunately, despite the various benefits of MSA, there is still a lack of systematic guidelines for microservice boundary identification in general [18]. Thus, it would be more feasible and practical to narrow our focus down to specific problems and reach consensus on concrete microservices in the same context. In turn, the predefined microservices can be shared and employed to facilitate constructing MSA-based applications in the specific problem domain. In other words, given a particular problem context, it will be worthwhile and beneficial to build a scaffold-like platform in advance by designing a family of domain-specific microservices (or microservice templates) that predefine different pieces of business logics potentially involved in the problem.

In this work, we dedicate ourselves to developing a microservice-oriented platform for IoBDA: i.e., the problem context here is IoBDA. As mentioned previously, in contrast to different instances of SDI, our platform emphasizes the microservice-level abstraction of IoT-friendly BDA logics. The so-called microservices contained in the platform essentially cater loosely coupled and distributable tasks of those BDA logics, as demonstrated in Section 4 and Section 5. Ideally, this context-specific platform will be able to cover various BDA logics, and each logic should have been well-analyzed into suitable tasks to match the predesigned microservices (and/or microservice templates).

Since there is no one-size-fits-all approach to BDA implementations, we are gradually investigating different data processing logics that are applicable to the IoT environment, such as Monte Carlo analytics, which can obtain approximate results through random observations or sampling, and Convergence analytics, which can rely on cascade MapReduce processes to gather increasingly dense results. It is noteworthy that both scenarios are compatible with the analytics characteristics in IoT, i.e., to divide a problem into micro-portions and to reduce unnecessary data transfer without sacrificing meaningful insights gained from the whole data [4,5].

In practice, the microservice-oriented platform will enable the implementation of data processing logics in the IoT environment via the orchestration of a set of standard microservices (and/or microservice templates). Benefiting from the nature of loose coupling, early-task microservices can be deployed close to where the data are generated so as both to avoid unnecessary data accumulation and to conduct data processing jobs more efficiently. In particular, programming a data processing logic will be realized as functional service calls without necessarily being concerned with or even being aware of the backend resource management.

More importantly, the microservice approach can conveniently address the challenges in the integration of heterogeneous sensor technologies [19,20] and in the communication between IoT sensors/devices. Similar to the early computer industry, today’s sensor industry has not yet employed or reached any global agreement on standard protocols and universal interfaces [21]. Given the various niche markets, different sensor vendors even tend to continue the existing incompatible protocols and keep their own proprietary standards in order to secure market shares against their competitors. By wrapping different data processing tasks into microservices, sensors will be able to expose their interfaces as unified and language-neutral APIs (e.g., typical REST APIs look like “cloudpracticeaesr.appspot.com/mci/1000000”, as demonstrated in Section 4), while diverse sensor communications will then become standard microservice communications that mainly rely on the predominant TCP/UDP-based Internet protocols [22].

## 4. Case I: Microservice-Oriented Logic for Monte Carlo Analytics

### 4.1. Architectural Design with Microservice Identification

**Figure 1:**
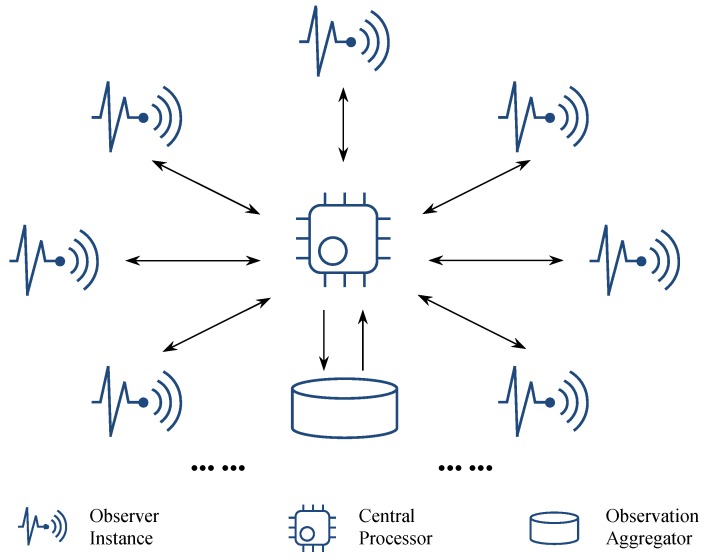
Star topology of the microservice-oriented logic for Monte Carlo analytics.

By stressing the independence and distribution of IoT sensors/devices, we came up with a microservice-oriented star-topology logic (cf. Figure 1) for implementing IoT-based Monte Carlo solutions for random sampling problems that can represent a broad class of easy-to-parallel applications. To address a random sampling problem is to statistically perform a set of observations from a tremendous range of (or even infinite) possibilities to approximate the answer under a particular condition. Following the Monte Carlo method, suppose the exact probability of a situation is *P*, and we randomly perform observations; then, the percentage of observations that match the situation will approximately be equal to *P* if the observation number is large enough. In this case, the workload of performing observations (e.g., population estimation through traffic monitoring in a city) can conveniently be divided into any size of pieces and distributed widely.

We are mainly concerned with three roles in the generic logic of Monte Carlo analytics, as illustrated in Figure 1. Correspondingly, we predefine microservices with respect to these roles and briefly explain them as follows.

- **Observer** is a microservice template to be instantiated for specific observation tasks. Multiple observation tasks can be accomplished either by a group of observer instances or by multiple observation activities of a single observer instance. As the name suggests, observer instances are supposed to be deployed or migrated to virtualization-friendly IoT sensors/devices or their governors.
- **Central Processor** splits a whole job into independent pieces as observation tasks, assigns individual tasks to available observer instances, and correspondingly receives observation results. In addition to passing the observation results to **Observation Aggregator**, the central processor can also incrementally retrieve and manipulate the aggregated observation results into continuous and value-added outputs if needed.
- **Observation Aggregator** collects, stores, and can directly output observation results if they are immediately ready to use. Note that the observation results here should not be a simple transition of original data from the observer side. Taking a sampling task as an example, the observation will send back a statistical result instead of the information about detailed samples (see the demonstration in Section 4.2). In other words, the observation aggregation here does not conflict with the principle of collectionless analytics in the IoT environment.

Given these predefined microservices and the pre-settled Monte Carlo processing logic, an IoT-friendly random sampling problem would quickly be addressed on such a platform. Note that the underlying resource management mechanisms (e.g., Google App Engine mentioned in Section 4.2) are completely transparent to the platform users.

### 4.2. Conceptual Validation Using Double Integral Estimation

To demonstrate and initially validate the effectiveness and efficiency of such a platform for Monte Carlo analytics, we selected the approximation of a double integral as the random sampling problem. A double integral of a function can be interpreted as the volume between the surface given by the function and its x–y region. In particular, the area of any x–y region that is represented as a single integral can also be estimated through the Monte Carlo method, as described by Equation (1).

(1) $$\int_{a}^{b}f\left(x\right)dx\approx \frac{b-a}{N}\sum_{i=1}^{N}f\left(x_{i}\right)$$

where $f(x_{i})$ is the *i*th random evaluation of the function within the interval [a,b], and *N* indicates the total number of trials in the Monte Carlo estimation.

Although the generic description in Equation (1) aims at a single integral, we can conveniently extend it to a double integral by iterating two similar steps, i.e., (1) area approximation and (2) volume approximation according to the estimated area, as concisely outlined in Algorithm 1.

**Algorithm 1** Monte Carlo Approximation of Double Integral
     **Input:** N: the total number of trials in Monte Carlo estimation.
     **Output:** The approximation result of the predefined double integral $\int_{a}^{b}\int_{g(x)}^{h(x)}f\left(x,y\right)dydx$.
1:valid_points←0                       ▹ for counting the number of points inside the valid x–y region.2:function_evaluations←0                       ▹ for summing up individual evaluations of f(x,y).3:X← a random number between *a* and *b*4:Y← a random number between MIN{g(x)} and MAX{h(x)}, x∈[a,b]5:
**for** i←1,N **do**
6:    **if** g(X)<=Y and Y<=h(X) **then**            ▹ If the random point (X,Y) is located inside the valid x–y region.7:        valid_points←valid_points+18:        function_evaluations←function_evaluations+f(X,Y)9:    **end if**10:
**end for**
11:*area*←(b-a)·(MAX{h(x)}-MIN{g(x)})·valid_points/N, x∈[a,b]  ▹ Estimating the area of the valid x–y region.12:volume←area·function_evaluations/valid_points      ▹ Note that the function was evaluated *valid_points* times.13:**return***volume*                              ▹ The estimated volume as the double integral result.


**Figure 2:**
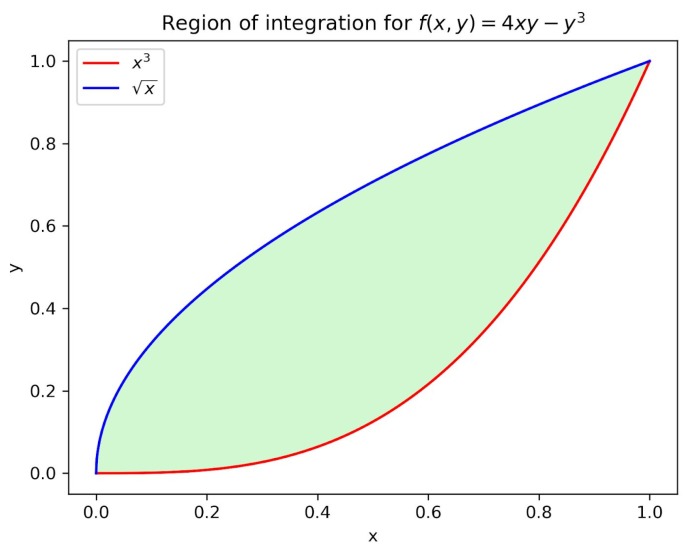
Projection of the function $f\left(x,y\right)=\left(4xy-y^{3}\right)$ onto the x–y plane, $x\in \left[0,1\right],\;y\in \left[x^{3},\sqrt{x}\right]$.

We explain the complete process by using a concrete example specified in Equation (2). The projection of the corresponding function $f\left(x,y\right)=\left(4xy-y^{3}\right)$ onto the x–y plane is illustrated in Figure 2. It is noteworthy that the x–y plane covering the projection happens to be a unit square in this case, and it is constrained by $x\in \left[0,1\right],\;y\in \left[x^{3},\sqrt{x}\right]$.

(2) $$\int_{0}^{1}\int_{x^{3}}^{\sqrt{x}}\left(4xy-y^{3}\right)dydx$$

When it comes to the area approximation, imagine that we blindly draw points inside the unit square; there must be some points drawn inside the valid x–y region of the projection and others that are not. After spreading numerous points randomly over the square, its area can be replaced with the number of points, as can the x–y region’s area. Then, we will be able to use the ratio of point numbers to satisfy the Monte Carlo approximation of the projection area, i.e., ${\mathrm{Amount}}_{\mathrm{projection}}/{\mathrm{Amount}}_{\mathrm{all}}={\mathrm{Area}}_{\mathrm{projection}}/{\mathrm{Area}}_{\mathrm{all}}$.

While drawing points, the evaluations of f(x,y) also accumulate at the points located inside the valid x–y region. Once the projection area is estimated, we can use the accumulated evaluations to calculate and sum up the corresponding solid volumes and eventually use the average volume to approximate the double integral result.

**Figure 3:**
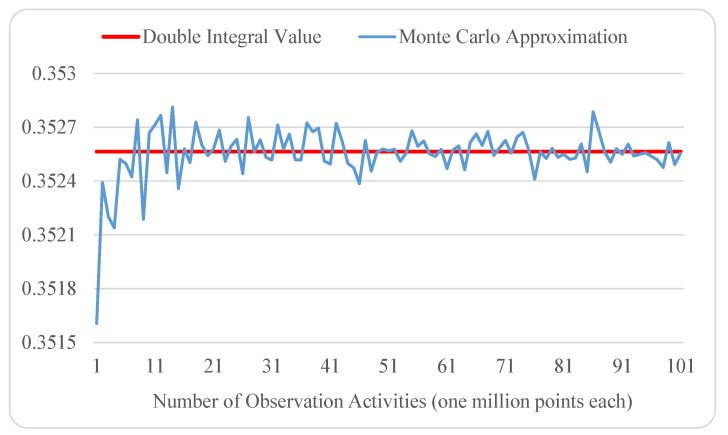
Monte Carlo approximation of the double integral $\int_{0}^{1}\int_{x^{3}}^{\sqrt{x}}\left(4xy-y^{3}\right)dydx$ as the sampling size (number of observation activities) grows.

Recall that, in general, the larger the number of random samples, the more accurate the Monte Carlo approximation. Here, we decided to split this unlimited size of sampling workload into individual tasks and define each task as drawing 1 million points in a unit square, i.e., x,y∈[0,1]. Then, we implemented the aforementioned observer role to fulfill such a task and deployed a number of observer instances on multiple Python runtimes of Google App Engine (one of the observer instances is at https://cloudpracticeaesr.appspot.com/mci/1000000, which returns (1) the number of points that fall inside the projection area from 1 million random-point generations and (2) the accumulated evaluations of the function $f\left(x,y\right)=\left(4xy-y^{3}\right)$ at those points). The experimental result of this Monte Carlo analytics experiment is visualized in Figure 3. Compared with the double integral value 55/156 or 0.3525641, it is clear that as the number of observer instances (or observation activities) grows, we can expect progressively more precise approximations of the double integral. Meanwhile, the local computing workload of the central processor will increase trivially, as it only deals with the random sampling results.

To help validate the potential efficiency of Monte Carlo analytics in the distributed IoT environment, we further conducted experiments to compare the performance of finishing the same integral approximation job (on the basis of 10 million random points generated) between the local centralized calculation and the application of different numbers of observer instances. In particular, our local environment is a laptop with an Intel Core i3-6100U @2.3 GHz processor and 4 GB of RAM. To simulate the distributed locations of sensors, we randomly deployed observer instances to different geographical regions of Google App Engine [23], as shown in Table 1.

**Figure 4:**
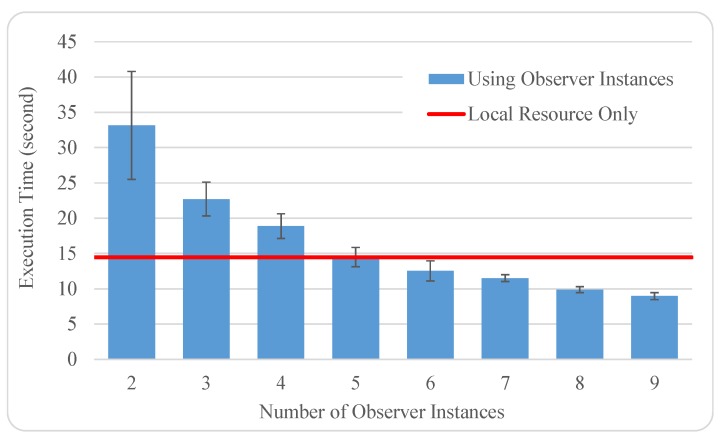
Monte Carlo approximation of the double integral $\int_{0}^{1}\int_{x^{3}}^{\sqrt{x}}\left(4xy-y^{3}\right)dydx$, with the fixed workload (10 million sampling points) as the number of observer instances growing. The error bars indicate the performance variations in the Monte Carlo approximation job.

By manually balancing the workload among observer instances and triggering multiple observation activities if needed, we obtained experimental the results illustrated in Figure 4. Note that all the experiments were repeated 10 times so that we could expect relatively more accurate results by using the average values. We also calculated the standard deviation of the 10-trial results from every experimental scenario, as indicated via the error bars in Figure 4. It seems that fewer observer instances incur higher performance variations. This phenomenon is due to fewer observer instances undertaking more observation activities to accomplish the same-sized job and, consequently, exaggerating the uncertainty in data transmission latency across the Internet.

Acting as a baseline for comparison, the average execution time of locally approximating the double integral $\int_{0}^{1}\int_{x^{3}}^{\sqrt{x}}\left(4xy-y^{3}\right)dydx$ (without considering any data transmission) is about 14.453 s. Since it does not make practical sense to compare the baseline with the case of a single-observer instance, we intentionally removed the single-observer performance from Figure 4. Overall, it can be seen that the Star-topology Monte Carlo analytics can beat its local version after involving more than five observer instances in this case, even though the distributed “sensors” require extra Internet communication overheads. In fact, there could be an even higher networking cost if collecting all the raw data together before analytics (cf. Section 5.2). Thus, the scenario of IoT-based Monte Carlo analytics would particularly be suitable for the applications when assuming “the more sensors, the better performance”, which essentially takes advantage of horizontally scaling the whole system. Such an advantage is also one of the reasons that we chose MSA to fit IoBDA, i.e., the microservice technology naturally matches the characteristics of IoT, particularly in terms of better scalability and maintainability [24].

### 4.3. Prospect of Practical Application

On the basis of this proof of concept for Monte Carlo analytics, we expect to prototype the platform by aligning with practical use cases. For example, a possible use case would be a traffic monitoring system that can facilitate urban design or help adjust local traffic policies. In detail, a single task to be fulfilled by the Observer will be counting vehicles and pedestrians (analogous to the points inside and outside the projection area, respectively, in Figure 2). The distributed observer instances can be deployed together with sensors at different crossroads. Then, our platform prototype with suitable central processing functionalities will be able to monitor and analyze the traffic information of a city.

By emphasizing the Star topology of data processing in IoT, the Monte Carlo logic can be conveniently extended to broader application types as long as the applications can take advantage of the aforementioned horizontal scalability. We take sorting as a generic example application other than Monte Carlo analytics. Imagine a job is to sort a large total of random numbers. The facilities of our platform (e.g., the possible Docker files and deployment routines) for supporting Monte Carlo analytics will remain the same, while developers only need to adapt the job’s implementation to the Star-topology architecture and instantialize the corresponding microservice templates. For example, a single task deployed in an observer instance can be (1) generating a small group of random numbers and (2) using the Quick Sort algorithm to sort the generated random numbers; the central processor can implement the Merge Sort algorithm to gradually receive and sort the numbers from all the observer instances.

## 5. Case II: Microservice-Oriented Logic for Convergence Analytics

### 5.1. Architectural Design with Microservice Identification

**Figure 5:**
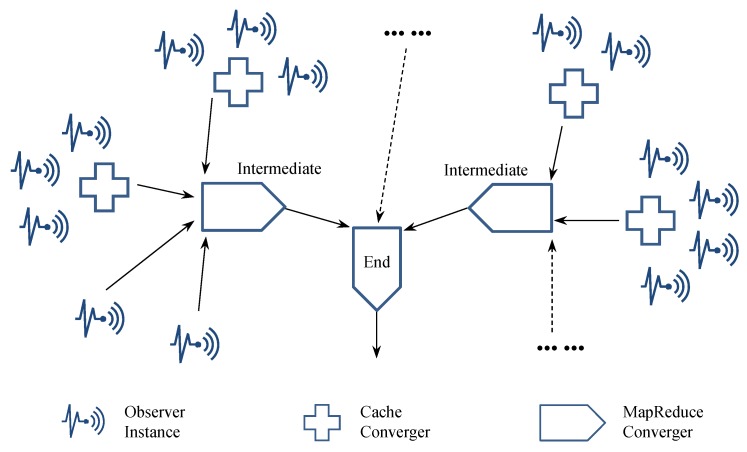
Tree topology of the microservice-oriented logic for Convergence analytics.

At this current stage, we mainly use the well-known MapReduce logic to represent Convergence analytics, which can significantly reduce the data size/amount during analytical processing, e.g., by merging original and intermediate data. Furthermore, to better fit the characteristics of IoT, we consider a Tree topology of Convergence analytics with cascade MapReduce logic in practice, as illustrated in Figure 5. From the perspective of topology, Convergence analytics can be viewed as an extension of Monte Carlo analytics. However, we distinguish between Monte Carlo analytics and Convergence analytics by emphasizing different concerns of collectionless BDA. Specifically, Monte Carlo analytics focuses on spreading observations, while Convergence analytics focuses on reducing the size of data transmission.

Similarly, we identify three main roles in Convergence analytics, as shown in the legends of Figure 5. For the purpose of conciseness, although the MapReduce logic can further be broken down into more specific roles (i.e., mapper and reducer) to be implemented as microservices, we do not elaborate on those well-known component roles here. In addition, to avoid duplication, there is no need to respecify the reusable role Observer (cf. Section 4.1). Thus, we only focus on and explain different convergers as follows.

- **Cache Converger** prepares data blocks by merging small pieces of data from a limited range/cluster of IoT sensors/devices, whereas it does not reduce the overall data size. Cache convergers could particularly be helpful for passing a large number of discrete data records to the subsequent MapReduce logic, as dealing with tiny-data transactions would be inefficient in terms of both execution time and energy expense [25]. In fact, caching data before transmission has become an energy optimization strategy, especially for mobile devices [26]. Note that cache convergers should be located at (or at least close to) the Internet edge in order to take advantage of the relatively trivial overhead of edge communication for receiving small data pieces.
- **Intermediate MapReduce Converger** either receives preprocessed data blocks from observer instances and cache convergers or receives pre-converged data from antecedent (also intermediate) MapReduce convergers and then uses the MapReduce mechanism to further converge the received data. Since we do not expect cache convergers to reduce data size/amount tremendously, the outermost MapReduce convergers should also be located close to the edge of the Internet.
- **End MapReduce Converger** receives final-stage intermediate convergence results and still uses the MapReduce mechanism to complete the whole analytics job. In contrast, the end MapReduce converger can be located remotely from the Internet edge. There is no doubt that the region-wide and cross-region communications will incur increasingly higher overhead; however, here we can expect to transfer less data as compensation, because the intermediate convergence results should have been much smaller than the sum of their raw inputs.

Note that, in practice, the Tree topology of a cascade convergence logic can be more flexible and comprehensive than that illustrated in Figure 5. As mentioned above, it is possible to plug and play more intermediate MapReduce convergers in series and/or parallel connections to conduct iterative Convergence analytics if needed for various workload distributions.

### 5.2. Conceptual Validation Using Word Count with Cascade Convergence

Here, we employ Word Count, which is the most popular MapReduce demo, to conceptually validate the microservice-oriented cascade convergence logic. Imagine that the requirement is to count words in a distributed text retrieval scenario, including numerous voice-to-text recognition and optical character recognition (OCR) sensors. Instead of transferring all the recognized texts to a central repository, the word counting job can be done on our platform with cascade convergers (cf. Figure 5). In particular, each cache converger can be implemented as a microservice which joins distributed “words” into a single “file” (i.e., a text document) and meanwhile performs simple data preprocessing or initial convergence tasks (e.g., restructuring data into predefined formats like JSON or XML). As for MapReduce convergers, the predesigned mapper/reducer microservice templates can be instantiated with the word-count-related functionalities and then be deployed/migrated to different and proper processing locations. The whole process of counting words through cascade convergence is specified in Algorithm 2. Note that the individual <word, 1> pairs are supposed to be manipulated when caching the input data (cf. the function Pairify(*S*) in Algorithm 2), which enables the unification of the Map procedures of all the MapReduce convergers, including the outermost one.

To facilitate monitoring of the changes in data size during the cascade convergence process, we conducted several rounds of word count experiments in our local environment. In particular, we replaced data caching with preparing a set of text files (ranging from around 2 MB to around 20 MB) by copying, pasting, and intentionally duplicating large amounts of random Wikipedia contents. As mentioned previously, the texts in each file were further restructured into <word, 1> pairs before going through the MapReduce convergers. When it comes to the MapReduce convergers, we employed three intermediate ones to imitate the outermost MapReduce processes within three different sensor regions so as to make the tree topology here consistent with the demonstration in Figure 5.

**Algorithm 2** Cascade-Convergence-based Word Count
     **Input:** S: the continuous string-format “sensor” data.
     **Output:** The word count result.
1:**function**Pairify(*S*)2:    P←∅3:    **for each** word w∈S **do**                    ▹ Restructuring data into <key, value> pairs and storing them.4:        P←P+<w,1>5:    **end for**6:    **return** *P*7:
**end function**
8: 9:**function**MapReduce(*F* list)10:    **procedure** Map(*F*)11:        **for each** line l∈F **do**                             ▹ Splitting the file into <key, value> pairs.12:           Parse *l* into <w,v>13:           EmitIntermediate(<w,v>)14:        **end for**15:    **end procedure**16:    **procedure** Reduce(*key, value_array*)17:        value_new←018:        **for each** value v∈ *value_array* **do**                      ▹ Counting the number of a particular word *key*.19:           value_new←value_new+v20:        **end for**21:        Emit(<key,value_new>)22:    **end procedure**23:    **return** <word,count> list24:
**end function**
25: 26:**while** receiving *S* **do**27:    **repeat**28:        F←∅                                        ▹*F* is the data block to be cached.29:        **repeat**30:           F←F+ Pairify(*S*)31:        **until** reaching the threshold size of data block32:    **until** having a *F* list33:    **for each** non-end MapReduce convergers **do**34:                                           ▹*F* list is for the outermost MapReduce convergers,35:                             ▹ while *intermediateResult* list is for the other intermediate MapReduce convergers.36:        *intermediateResult* ←MapReduce(*F* list or *intermediateResult* list)37:    **end for**38:
**end while**
39:result←MapReduce(final *intermediateResult* list)            ▹ Delivering final result by the end MapReduce converger.40:
**return** *result*



**Figure 6:**
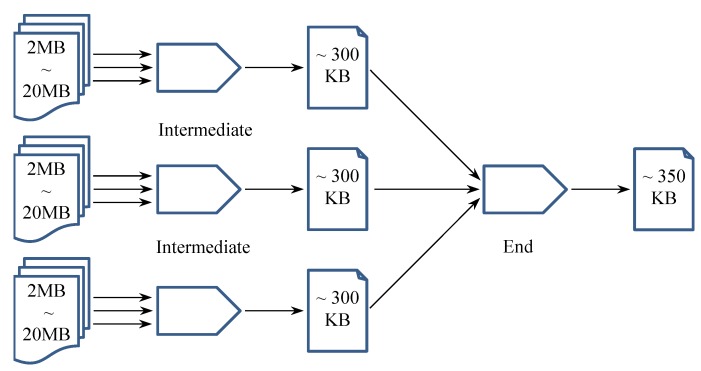
Data size changes during the cascade convergence process of word count experiments.

Since different experimental results vary significantly because of different sizes of professional vocabularies in the input files (e.g., same-field texts vs. multi-field texts), we only show the rough data size changes as an average during the MapReduce convergence process, as portrayed in Figure 6.

**Figure 7:**
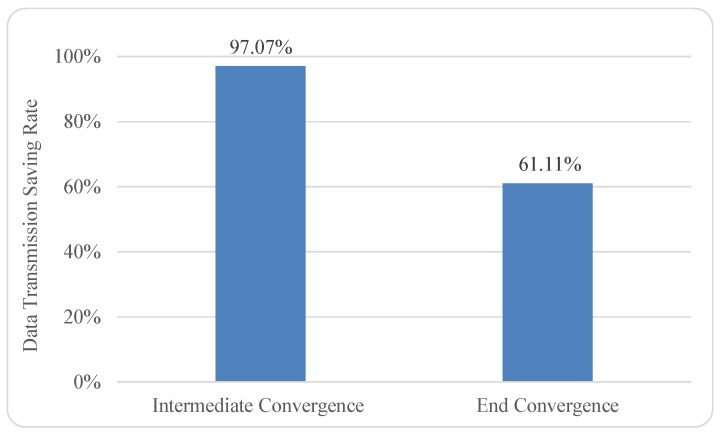
Data transmission saving rates of the intermediate convergence process and the end convergence process in the Word Count experiment.

It is clear that by tremendously reducing the data size through the outermost (as well as intermediate) MapReduce convergers, the rest of the convergence process enjoys much less overhead for data transmission. We further define the data transmission saving rate as a metric to quantitatively investigate the efficiency of Convergence analytics, as specified in Equation (3).

(3) $$R=\frac{D_{in}-D_{out}}{D_{in}}\times 100\%$$

where *R* represents the data transmission saving rate of a particular convergence process (by any type of converger). $D_{in}$ and $D_{out}$ respectively indicate the input data size before the convergence process and the output data size for the subsequent transmission. After applying this metric to our Word Count experiment, we illustrate the quantitative measurement in Figure 7. Note that, for the purpose of conciseness, we used 10 MB as the representative size of input data (i.e., the prepared text files ranging from around 2 MB to around 20 MB) for the intermediate convergence process to calculate its data transmission saving rate.

Specifically, the data transmission saving rates of intermediate convergence process and end convergence process within our experiment are roughly 97.07% and 61.11%, respectively. We believe that such high rates are the result of the extreme case of counting words, because the vocabulary size of human languages is fairly small, especially at the conversational level [27]. We reckon that other applications of Convergence analytics could have more moderate rates of data transmission saving.

### 5.3. Prospect of Practical Application

On the basis of the same logic of the cascade word count, we expect to solidify our microservice-oriented platform with respect to Convergence analytics through a real project, i.e., visualizing large spatial datasets in a Web-based map viewer [28], which requires handling tons of position events generated by GPS devices (vehicles) and quickly visualizing integration results at the client side, especially when switching zoom levels.

Similarly, without changing the Tree topology of data processing in IoT, our proposed platform can naturally support more application types beyond Convergence analytics. Still taking sorting as an example, we can reuse the same architecture (cf. Figure 5) and microservices (and templates) to quickly satisfy such a different job. In detail, the cache convergers can directly be reused to collect numbers from nearby sensors and pass data blocks to the subsequent MapReduce convergers, and the MapReduce convergers should be equipped with suitable sorting functionalities [29]. Note that, benefiting from the cascade convergence logic, users will not have to rely on a single MapReduce converger with heavyweight implementations (e.g., what Google did includes thousands of nodes [29]).

## 6. Conclusions and Future Work

Different types of SDI, such as SDN and SDS, have widely been accepted to make physical computing environments programmable. In essence, the idea of SDI is still from the computing resource’s perspective. In contrast, we propose a microservice-oriented platform from the application’s perspective in order to help make domain-specific business logics microservice-able and further decouple business logic implementations from resource control and management. Considering the tremendous distribution of IoT data, such a microservice-oriented platform will particularly be valuable and useful in the IoBDA domain by relieving the complexity in and breaking the environmental monolith of traditional BDA mechanisms.

In summary, we use this article to initialize an ambitious proposal at its proof-of-concept stage. Although it is impossible (and unnecessary) to address all kinds of BDA problems, we have shown that suitable scenarios such as Monte Carlo analytics and Convergence analytics in IoT can benefit from the proposed microservice-oriented platform. We plan to follow a reinforcement process to develop this platform for IoBDA. The platform prototype will stick to limited data processing logics and applications and then use new applications and include new BDA logics to gradually validate, improve, and enrich the platform features.

Thus, our future work will unfold along two directions. Firstly, we will try to extend the two conceptual validation demos described in this article to more practical case studies. Considering the relatively high failure ratio and long latency from IoT sensors/devices, we will particularly focus on investigating fault-tolerant mechanisms within the practical IoT environment. In fact, it has been identified that MSA can naturally help isolate failures [30] and increase application resilience [31], i.e., one failed microservice does not necessarily affect the other microservices, at least when there is no chaining between them. Secondly, we will keep enriching this proposed platform, e.g., including MSA-friendly machine learning logics [32]. By integrating more types of data processing logics, the eventually developed platform will provide a catalog of IoBDA scenarios together with demo applications, which can guide users to select suitable microservices and instantialize relevant microservice templates for their specific IoBDA implementations.

## Figures and Tables

**Table 1:** Multiple observer instances randomly deployed to different regions of Google App Engine.

Number of Deployed Observer Instances	Region	Number of Deployed Observer Instances	Region
Two	us-central (Iowa)	One	us-east1 (South Carolina)
One	us-west2 (Los Angeles)	One	us-east4 (Northern Virginia)
One	northamerica-northeast1 (Montréal)	One	southamerica-east1 (São Paulo)
One	europe-west2 (London)	One	europe-west3 (Frankfurt)

## Abbreviations

The following abbreviations are used in this manuscript:

API	Application Program Interface
BDA	Big Data Analytics
IoBDA	Internet of Big Data Analytics
IoT	Internet of Things
JSON	JavaScript Object Notation
MSA	Microservices Architecture
OCR	Optical Character Recognition
REST	Representational State Transfer
SDI	Software-Defined Infrastructure
SDN	Software-Defined Network
SDS	Software-Defined Storage
XML	Extensible Markup Language
